# Editorial

**Published:** 1853-11

**Authors:** 


### THE MEDICAL EXAMINER.

PHILADELPHIA, NOVEMBER, 1853.

### YELLOW FEVER IN PHILADELPHIA.

It is with much pleasure that we announce to our readers that the Yellow or Malignant fever has entirely disappeared from our city; no new cases having occurred since the 7th of October. Nor is it with less satisfaction that we communicate the fact, that at no period of the year has our city enjoyed a greater degree of health than at the present time.

The whole number of cases of Malignant fever that have occurred since the 19th of July, as far as we have been able to collect them, have been one hundred and seventy. These embrace all reported to the Board of Health, together with a few others, that did not come under their notice. They include every variety of the disease, or those recognized in the nomenclature, as Yellow, Malignant, Malignant Bilious, Pernicious, Malignant Remittent, and Typhus Icterodes fevers.

One hundred and forty-seven of these cases may be distinctly traced to the immediate vicinity of the infected district, making South Street wharf the centre. Of the remainder it is uncertain where the fever was contracted.

The ratio of mortality has been very small, compared to the whole population of that section of the city where the disease prevailed; but, when calculated by the number of persons attacked, it has been proportionably large.

Out of 170 cases there were 128 deaths, equal to 75 per cent. or one death in every 1.42 hundreths.

This fatality, must be ascribed to the virulence of the atmospherical poison, and the character and habits of many of those who were exposed to its morbific influence. It must not, however, be entirely overlooked, that there have been other cases of fever in the infected locality, assuming a less malignant form, which being readily cured, were not considered by their medical attendants as suitable cases to report to the Board of Health. Had all such been included in the above estimate, they would have increased the number while they would have materially diminished the ratio of mortality, and at the same time have placed the success of the treatment, in a much more favorable aspect.

The locality of the fever has been very much circumscribed, its ravages having been confined to narrow limits. Union street on the north, Second street on the west, Queen street on the south, and the Delaware river on the east, may be set down as its boundaries, although we have the record of a few isolated cases, originating beyond these limits, between which and the infected locality, we could not trace any connexion.

During the interval between the date of the first case of Malignant Fever, July 19th, and the last, October 7th, including a period of eighty days, it is doubtful, when we compare the quarterly statistics of mortality, published in this number, with those of similar periods in preceding years, whether the health of the city in general has suffered any material deterioration. For instance, in 1852 the deaths for the quarter ending October 2nd, class Zymotic or Epidemic, were 980, among which fevers are included. This year they were 999, being an increase of only nineteen over those of the former, although malignant fever prevailed.

Notwithstanding, therefore, all the hue and cry among alarmists at home, and those envious of our reputation for health abroad, about the extent of the disease and its fearful ravages, we may safely assert, that the general health of Philadelphia as well as its increasing commercial interests, have not experienced any interruption.

Among all the cases of Malignant fever that have happened this season in our city, of which we have any record, there is not a single instance of its occurrence among the colored population. This fact is in accordance with a generally received observation, that the African race is much less susceptible to malarious influences than others.

Another circumstance worthy of record is, its non-contagiousness. This must be the uniform evidence of all who have had anything to do with the disease. Not an instance can be cited of its having reproduced itself. Many of the cases where the fever was contracted by a visit or a residence within the infected locality, were treated far beyond the limits, and in different parts of the city, without communicating the disease to others.

In the Pennsylvania Hospital 24 cases were treated, and in wards surrounded by those who were sick and convalescing from other diseases, and where there was continual intercourse with the patients, and not an individual in the house was attacked with the fever. The like immunity was observed at the Blockley hospital, where, had the disease possessed contagious qualities, it would have been very likely to have spread among the inmates.

In conclusion we would add, that we are more than ever convinced of the importance of sanitary measures in a large city. Our experience during the past few months has demonstrated their power and value. We are confident that judiciously administered sanitary operations are all that are required to avert the malignancy and fatality of epidemics. Cleanliness, pure air, and health go hand in hand. While filth, an impure atmosphere, and disease, are universally concomitants, one never being found without the other.

In a salubrious climate like that of Philadelphia, we should have no excuse for the spread of an epidemic. There do exist, it is true, as in all cities, where the laws of Hygiene have not been studied in the laying out of streets and in the erection of dwellings, numerous localizing causes, favorable to the outbreak and spread of epidemics, but by enforcing an early and energetic system of sanitary appliances, much may be done by the custodians of the health of the city, towards mitigating and arresting the spread of disease. W. J.

